# Energy-Efficient Opportunistic Routing Algorithm for Post-Disaster Mine Internet of Things Networks

**DOI:** 10.3390/s23167213

**Published:** 2023-08-16

**Authors:** Qing Zhao, Wei Yang, Liya Zhang

**Affiliations:** 1 School of Electronic and Information Engineering, Beijing Jiaotong University, Beijing 100044, China; wyang@bjtu.edu.cn (W.Y.); 20111076@bjtu.edu.cn (L.Z.); 2 China Coal Research Institute, Beijing 100013, China

**Keywords:** Mine Internet of Things (MIoT), post-disaster reconstruction, opportunistic routing (OR), data transmission, energy efficient, routing void

## Abstract

The Mine Internet of Things (MIoT), as a key technology for reconstructing post-disaster communication networks, enables a user to monitor and control the safety of an affected roadway. However, due to the challenging underground mine environment, the MIoT suffers from severe signal attenuation, vulnerable nodes, and limited energy, which result in a low level of network reliability for the post-disaster MIoT. To improve transmission reliability and reduce energy consumption, a directional-area-forwarding-based energy-efficient opportunistic routing (DEOR) approach for the post-disaster MIoT is proposed. DEOR defines a forwarding zone (FZ) for each node to route packets toward the sink. The candidate forwarding set (CFS) is constructed by the nodes within the FZ that satisfy the energy constraint and the neighboring node degree constraint. The nodes in the CFS are prioritized based on a routing quality evaluation, which takes the local attributes of the nodes, such as the directional angle, transmission distance, and residual energy, into consideration. DEOR adopts a recovery mechanism to address the issue of void nodes. The simulation results verify that the proposed DEOR approach outperforms the ORR, OBRN and ECSOR methods in terms of energy consumption, average hop count, packet delivery rate, and network lifetime.

## 1. Introduction

**Figure 1:**
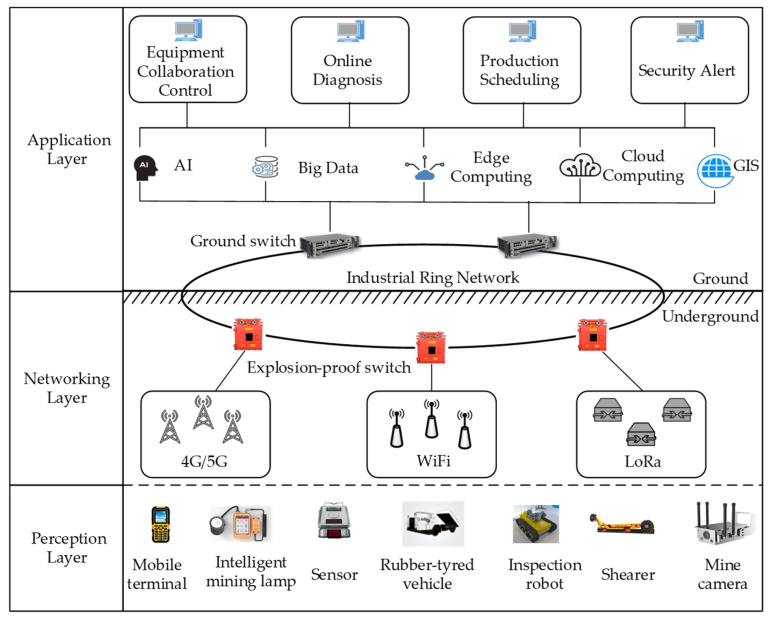
Three-layer architecture of a typical MIoT system.

The Mine Internet of Things (MIoT) has become a widely used technique that achieves the construction of smart mines by employing IoT technologies in industrial mining scenarios [1]. Generally, the system architecture of the MIoT is composed of three layers, i.e., a perception layer, a network layer, and an application layer, as shown in Figure 1. The perception layer is the foundation of MIoT applications, consisting of various intelligent terminals and devices equipped with sensors that are responsible for collecting physical information, such as data regarding personnel, equipment, and the environment in the mine. The information collected by the perception layer is transmitted to the application layer via the network layer. At present, there are many wireless communication technologies available for data transmission in underground mines, such as4G/5G, Wi-Fi, and LoRa [2]. In the application layer, the collected information can be processed and analyzed, and appropriate decisions are made to meet different production requirements. The MIoT system has been utilized to monitor the status of an entire mine, thereby improving production safety and the level of accident prevention in the mine [3].

In the actual process of coal mining, disasters such as gas explosions, roof shedding, and water penetration can easily occur due to the harsh environmental conditions [4]. These disasters usually cause roadway blockages or personal injuries, etc., preventing underground personnel from actively evacuating the accident area and constraining them to wait for rescue in the mine roadway. However, rescuers entering the accident area after the disaster are prone to secondary accidents due to unknown information about the mine’s environment, triggering even more casualties. In order to access specific information about the disaster roadway, environment detection should be carried out for the affected area before the rescue [5]. In fact, some communication facilities may be damaged in accidents, leading to the interruption of original communication links and the loss of on-site information in post-disaster accident areas [6]. Consequently, restoring normal communication between the accident area and the ground command center as soon as possible after the disaster has become an urgent issue.

In general, post-disaster communications can be restored by laying cables or placing auxiliary communication devices in accident tunnels. However, this approach is costly to deploy and cannot be achieved in a short time underground in the mine [7]. To ensure timely and efficient underground communications, the surviving facilities can be utilized to quickly reconfigure the post-disaster network. In addition, post-disaster communications should be capable of perceiving environmental and personnel information and promptly transmit the site information from the incident area to the rescue center. Therefore, reconstruction of the Mine Internet of Things (MIoT) using wireless networks is an effective solution.

Considering a scenario in which the Mine Internet of Things (MIoT) is rescued in the context of a fully mechanized mining face accident, a large number of surviving nodes are randomly scattered in the accident area, and the planar topology is managed via self-organization to re-establish the post-disaster MIoT network based on a wireless multi-hop routing sensor network. These nodes have the ability to perceive and transmit data information. When the source node collects data information regarding the mine disaster, it uses a multi-hop routing data transmission method to forward the data packet to the sink node, thus completing the effective monitoring of the fully mechanized mining face. Unlike normal mining scenarios, the surviving nodes in post-disaster MIoT networks have extremely limited amounts of energy and cannot be replenished in a timely manner. In addition, frequent data transmission after disasters can cause high levels of communication energy consumption, thereby shortening the entire network’s lifetime [8]. Due to the collapse and dispersion of objects such as coal blocks, rock masses, and electrical devices, network nodes in the roadway can be damaged or displaced, which causes the topology to become sparse [9]. In addition, the affected roadway environment is highly complex, with a non-uniform, layered geological structure that conducts a large amount of signal loss and multipath attenuation. As a result, the high rate of packet loss will lead to the failure of the post-disaster MIoT to ensure the reliable transmission of data. Due to the aforementioned problems, existing wireless network routing protocols cannot be applied well to post-disaster MIoT data transmission. Therefore, an efficient and reliable routing protocol is urgently required for post-disaster MIoT.

Opportunity routing (OR) can improve the reliability of data transmission in wireless networks by fully utilizing the broadcasting characteristics of wireless channels and collaboration between forwarding nodes [10]. Initially, OR was designed to meet the communication requirements of ad hoc networks in sparse mobile scenarios. Nowadays, it has become an important technique for data collection and sharing in wireless multi-hop networks. Considering various Quality-of-Service (QoS) requirements for WSNs, a fuzzy-based load-balanced opportunity routing protocol is proposed [11]. In contrast to traditional deterministic wireless network routing protocols, OR does not require the sender to select a specific relay node before data forwarding, but only needs to maintain the candidate forwarding set (CFS). Moreover, OR can effectively address the problem of link unreliability caused by unstable wireless channels, thus reducing packet retransmissions and improving network throughput. In [12], a virtual-range-forwarding-based opportunistic routing is proposed to overcome the unfavorable characteristics of wireless channels. Hence, the OR method can effectively cope with the vulnerability and the intermittent connectivity of the post-disaster MIoT networks.

Nevertheless, there are some drawbacks to traditional OR research studies. On the one hand, the selection of routing metrics is relatively single, without considering various attributes of the network comprehensively. On the other hand, these protocols are designed to improve packet delivery success rates by using a large number of duplicate packets while ignoring the high latency problem in the network. In reality, high latency in MIoT scenarios can seriously affect the efficiency of accident rescue work. Different from the terrestrial routing method, there exist the following challenges for opportunistic routing in the post-disaster Mine Internet of Things (MIoT). First, the surviving nodes after the disaster are constrained by the limited power resources. If there are too many nodes participating in packet forwarding, high communication energy consumption will be generated in the network, and nodes will fail quickly due to energy depletion, thereby aggravating the intermittent connectivity of the post-disaster MIoT. Conversely, if there are few nodes for packet forwarding, the packet delivery success rate of the post-disaster MIoT cannot be guaranteed. Therefore, the size of the candidate forwarding set becomes a crucial factor affecting the network performance. Second, the MIoT network data transmission in affected regions tends to be directional, and critical network nodes close to the sink will undertake more transmission tasks, resulting in the routing hotspot problem, which severely reduces the energy utilization of surviving nodes. Third, the harsh environment in post-disaster underground mines makes surviving nodes prone to physical damage, causing unstable network topology and void routing, which severely degrade the reliability of transmission links.

To the best of our knowledge, there are fewer studies on OR methods in the post-disaster Mine Internet of Things (MIoT). Therefore, the focus of our work is to address the aforementioned problems of opportunistic routing in the post-disaster MIoT. In this paper, we propose a Directional-area-forwarding-based Energy-efficient Opportunistic Routing (DEOR) algorithm to improve the reliability and robustness of data transmission in post-disaster MIoT networks.

The main contributions and innovations of this paper are summarized as follows.

1. In order to restore post-disaster data transmission of MIoT in the planar accident area rescue scenarios, such as a fully mechanized coal face, a post-disaster flat network architecture of MIoT based on the multi-hop routing of surviving nodes is established, which consists of a sink node and multiple sensor nodes. This network architecture achieves the purpose of comprehensive perception and effective transmission of environmental information in the planar accident mine after disasters;
2. We propose a directional-area-forwarding-based candidate forwarding set construction strategy. In the network initialization phase, according to the deployment density and communication radius of nodes in the accident roadway, a forwarding zone (FZ) is designed for each node to route packets toward the sink. Then, the candidate forwarding set (CFS) is constructed by the nodes within the FZ that satisfy the energy constraint and the neighboring node degree constraint. By restricting the number of duplicated packets in the network, DEOR improves the energy utilization of the surviving nodes;
3. We propose a relay node selection method based on routing quality evaluation. In the data transmission phase, we take multiple attributes of the nodes into account, such as direction angle, transmission distance, and residual energy. Next, nodes in the CFS are prioritized based on the routing quality, and the forwarding node with the highest priority is selected as the relay node to forward packets. Other nodes in the CFS discard packets after listening for a successful transmission message. By utilizing the collaboration between forwarders, DEOR addresses the hotspot problem and balances the traffic load between nodes in the post-disaster network;
4. We design a recovery mechanism for void nodes. When packets encounter the routing void during forwarding, a recovery mechanism is triggered. By employing the modified routing quality evaluation function, packets can bypass the void routing region and select available relay nodes to continue forwarding. DEOR overcomes the void routing node problem and improves the robustness of the whole post-disaster network.

Extensive simulation tests proved that the proposed DEOR algorithm obviously improves the performance of post-disaster MIoT data transmission in a planar accident mine. Moreover, our work provides a novel data transmission method for emergency communication in coal mines, which is of great practical significance for improving the efficiency of post-disaster rescue.

The rest of this paper is organized as follows. Section 2 discusses the related works. In Section 3, we describe the system model and problem description. Section 4 explains the proposed DEOR algorithm. Section 5 elaborates on the simulation setup and simulation results. Finally, Section 6 concludes this paper.

## 2. Related Work

In this section, we mainly review the related work on Mine Internet of Things (MIoT) routing protocols and opportunistic routing protocols, respectively.

### 2.1. MIoT Routing Protocols

Here, we briefly present some existing well-known routing protocols for MIoT networks. A comparison of the protocols is listed in Table 1.

On the one hand, several routing protocols (e.g., [13,14,15]) were proposed to enhance the performance of data transmission in normal coal mine tunnels. Yu et al. [13] proposed a Routing Protocol in mine roadways based on Area Positive Clustering and Mobile Nodes (RPAPC-MN). In RPAPC-MN, an area-positive clustering mechanism is applied to avoid data re-entry. By utilizing nodes equipped with miners and mobile equipment to forward data, RPAPC-MN reduces energy consumption and extends the network lifetime. You et al. [14] proposed a Differential Evolution routing (DE) protocol for wireless multimedia sensor networks for underground mines. By setting different weights in the normalized sub-QoS evaluation function of transmission delay, packet loss rate, and transmission energy consumption and applying the marking method to obtain the transmission path, DE offers a differentiated service and guarantees the QoS for multimedia services. Wu et al. [15] proposed an Optimal Forwarding Algorithm (OFA) for coal mine WSNs. When sensor nodes are evenly deployed in narrow lanes, OFA can dynamically adjust the node’s forwarded data volume and forwarded routing to balance the node energy consumption and maximize the network life cycle. On the other hand, a number of routing protocols (e.g., [16,17,18,19,20]) were proposed to restore reliable data transmission in mine tunnels after disasters. Wu et al. [16] proposed a Self-organized Energy-efficient Clustering (SEC) protocol for post-disaster reconstructed MIoT networks. SEC designs the election probability function based on the energy factor and connectivity factor. In SEC, the cluster head (CH) node was replaced by periodically comparing the residual energy and the threshold energy, which reduced the overall energy consumption and improved the stability of reconfiguration networks. On this basis, Zhao et al. [17] proposed an Energy-balanced Adaptive Uneven Clustering (EAUC) to solve the issue of node energy heterogeneity after disasters. EAUC introduces the CH election threshold function according to residual energy, relative distance, and distribution density of surviving nodes. The weight function of the relay CH was built by considering the current residual energy of the relay CH, and the CH with the largest weight was selected as the next hop routing node. Hu et al. [18] proposed a post-disaster reconstruction algorithm of a wireless ad hoc network in coal mines based on the Multi-dimensional Virtual Backbone Network (MVBN). The MVBN constructs a comprehensive evaluation index of virtual backbone nodes by considering betweenness centrality, node closeness, and residual energy. In the MVBN, nodes with a large evaluation index are elected as dominant nodes, so as to enhance the robustness of the virtual backbone network and prolong the network’s lifetime. Hu et al. [19] proposed a Network Hole Coverage Reconstruction Algorithm with Obstacles (NHCRA-O) for post-disaster MIoT. In NHCRA-O, Delaunay triangulation is used to divide residual nodes and corner points of obstacles in the network. The virtual repair node position is determined through the distance between the centroid and the vertex of the Delaunay triangle. NHCRA-O selects CHs according to the residual energy factor, node connectivity and directional betweenness, which significantly extends the network lifespan. Chen [20] proposed an energy-balanced Routing protocol based on the Improved Ant Colony algorithm (RIAC) to address the problem of uneven energy consumption between network clusters after disasters. RIAC introduces residual energy, distance, and node trust factors based on the traditional ant colony algorithm. By modifying the transition probability formula, pheromone heuristic function, and pheromone update strategy, RIAC realizes the optimal path selection from the CH to the base station. However, the above routing protocols used for MIoT networks adopt a clustering approach with predefined routing paths, which ignores the intermittent connection problem of wireless communication links after disasters in the routing process. When sparse topology occurs in the network, these existing methods cannot guarantee reliable data transmission for post-disaster MIoT networks.

### 2.2. Opportunistic Routing Protocols

Opportunity Routing (OR) was proposed mainly to improve the packet delivery rate of wireless networks. The design of an OR protocol focuses on four aspects: (1) construction of the candidate forwarding set, (2) selection of relay nodes and forwarder coordination, (3) data transmission and identification, and (4) dynamic adaptation to network topology changes. Currently, a plethora of OR protocols have been designed for various application scenarios [21]. Sang et al. [22] proposed an opportunistic routing protocol based on trajectory prediction (EORB-TP) to deal with the dynamic topology and unstable links of the FANET. EORB-TP defines the trajectory metric value based on node movement distance, node direction, and node density. In EORB-TP, an energy-saving data forwarding strategy is designed to deal with the limited energy resources and storage space of UAVs. In [23], the Delay and Duplicate transmission Avoid CFS optimization algorithm (DDA) was proposed to reduce the transmission delay and duplicate transmission in opportunistic routing for wireless muti-hop networks. DDA divides the nodes in the CFS into different fully connected relay networks and chooses the most appropriate relay network based on the multi-attribution decision-making (MADM) theory. Zhao et al. [24] proposed an opportunistic routing algorithm based on trust relationships for wireless mesh networks (WMNs) to solve the problem of low message delivery rate and high network resource consumption when forwarding messages in opportunistic networks. Li et al. [25] proposed an efficient and reliable transmission power control-based opportunistic routing (ERTO) for wireless ad hoc networks to improve packet delivery probability and reduce energy consumption and network interference. In ERTO, the packet delivery probability, the expected energy consumption, and the relationship between transmission power and node degree are applied to optimize the transmission power and forwarding node degree jointly. Banyal et al. [26] proposed a Hierarchical learning-based sectionalized routing paradigm for pervasive communication and Resource efficiency (HiLSeR) in an opportunistic IoT network. HiLSeR enables message routing using a combination of controlled-parameterized flooding and opportunistic sector-based decentralized transmission. Celik et al. [27] proposed a Sector-based Opportunistic Routing (SectOR) protocol for hybrid underwater optic acoustic networks. SectOR uses a variety of local and global metrics to evaluate the fitness of a candidate set (CS) and develops candidate prioritization techniques for various OR metrics. Zhou et al. [28] proposed a Network Coding Combined with Link Correlation (NCCLC) method to improve the energy efficiency in wireless edge IoT applications. In NCCLC, the link correlation was considered when calculating the number of required forwarding and selecting the forwarder set. In [29], the Community clustering Routing protocol based on information Entropy in mobile opportunity Networks (CREN) was proposed to deal with the different characteristics between communities and the inefficient nodes after community clustering. CREN reduces the transmission delay and routing overhead. Gopal et al. [30] proposed the detection of selfish nodes in a dynamic ad hoc network (DANET) via employing evidence-based detection. An Improved Self-Centered Friendship (ISCF) tree was constructed using Trust Authority (TA) to deal with security issues. The proposed method performs well in terms of selfishness detection accuracy and selfishness detection time. In [31], a Communication Trust and Energy Aware (CTEA) routing protocol was proposed to mitigate the effects of bad mouth and energy drain attacks. CTEA increases the packet delivery ratio, residual energy, and network lifetime by mitigating the nodes’ misbehavior in the presence of energy drain and bad mouth attacks.

In contrast to the existing opportunistic routing approaches, the proposed DEOR is a non-cluster and non-predefined opportunistic routing algorithm for post-disaster MIoT networks, which aims to address the issue of intermittent connectivity of wireless transmission links caused by the failure of surviving nodes during post-disaster network data transmission in planar accident area rescue scenarios, such as a fully mechanized coal face. DEOR improves the energy efficiency of the post-disaster network by dynamically adjusting the number of the candidate forwarding nodes. Moreover, DEOR overcomes the void routing node problem and improves the robustness of the post-disaster network by selecting available relay nodes in a downward path to the sink.

## 3. System Model and Problem Description

### 3.1. Network Architecture

**Figure 2:**
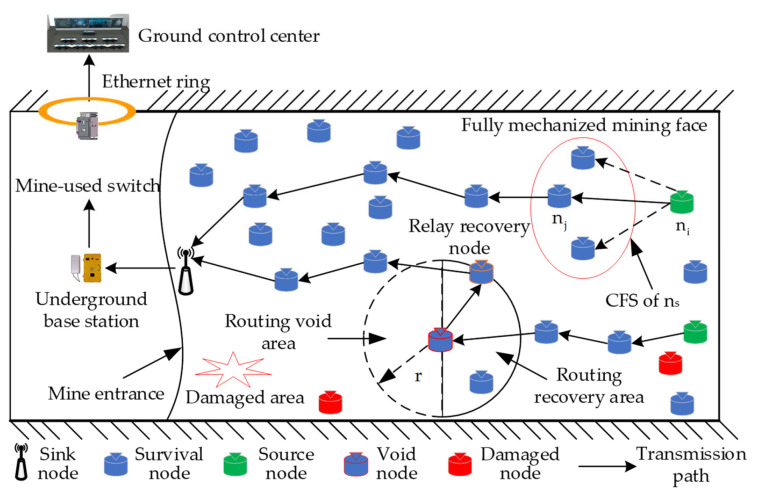
Opportunistic routing network model for post-disaster MIoT.

In the accident rescue communication scenario of planar areas such as fully mechanized coal mining working faces, we consider a network architecture for post-disaster Mine Internet of Things (MIoT) opportunity routing, as shown in Figure 2. The post-disaster MIoT network consists of two types of available nodes, i.e., multiple surviving sensor nodes and one sink node. All surviving nodes with the ability to sense and transmit information are randomly distributed in the accident tunnel. After the disaster, these sensor nodes are responsible for periodically collecting environmental and equipment information from the accident field. In addition, the sink node is deployed at the entrance of the accident mine and can adopt a wired power supply after the disaster, so the power is considered infinite. Before the post-disaster data transmission, the sink node will broadcast a detection message containing its own location information to the entire affected area with maximum transmit power. Then, source nodes in the network perform directional routing based on the sink’s location information, aiming to send data information to the destination node. As the gateway node of the post-disaster network, the sink is responsible for collecting various information collected by sensor nodes. In the post-disaster MIoT, each node can directly or multi-hop forward data packets to the sink node.

In the data transmission process of the post-disaster network shown in Figure 2, the source node $n_{i}$ that currently carries the data packet first constructs the CFS within its one-hop communication range for packet forwarding and sends the data packet to all neighbor nodes in the CFS to improve the success rate for packet forwarding. Then, a candidate node $n_{j}$ is selected as a relay node for $n_{i}$ through the node collaboration in the CFS. When the node $n_{j}$ successfully sends the packet to the next hop routing node, other candidate nodes in the CFS will drop the packet copies. According to this data transmission method, the sender carrying the data packet can select the appropriate relay node to forward the packet until the data information of the source node $n_{i}$ is sent to the sink. Next, the information is transmitted to the underground base station in the mine’s normal region through the sink. Finally, the information is sent to the ground control center through mining switches and Ethernet ring networks. Consequently, the post-disaster MIoT enables effective monitoring of the entire accident area through the proposed data transmission scheme.

We assume that all surviving nodes are homogeneous and stationary. Each node knows its geographical location through any re-localization scheme of drift nodes after mine disasters (e.g., the scheme studied in [32,33]), and the location of the sink is public. The post-disaster network is formed from a set of a finite number of surviving nodes, denoted by $N=\{n_{1},n_{2},\ldots ,n_{\left|N\right|}\}$. Any node $n_{i}\in N$ is located at $\left(x_{i},y_{i}\right)$, and has a limited communication range r and initial battery power $e_{\mathrm{ini}}$. For node $n_{i}$, its neighbor node set is denoted as $N_{i}=\{n_{j}|n_{j}\in N\;\&\;d_{i,j}\leq r\}$, where $N_{i}\subseteq N$ and $d_{i,j}$ denotes the Euclidean distance from $n_{i}$ to $n_{j}$. The length of the neighbor set is denoted by $m_{i}=|N_{i}|$. All nodes know their neighbors using any neighbor discovery strategy (e.g., the strategy studied in [34,35]), thereby calculating the distribution density of surviving nodes in the current accident area. In this paper, we define the data forwarding direction toward the sink in the underground accident region as an upward routing path, i.e., the forwarder is closer to the sink than the current sender. The notations used frequently in this paper are listed in Table 2.

### 3.2. Energy Model

Usually, the nodes in the Mine Internet of Things (MIoT) are sensor devices whose energy is limited and cannot be replenished after a disaster. In this section, we adopt the first-order radio energy consumption model to calculate the energy dissipation of the surviving network nodes [36]. In the MIoT, the energy cost of data transmission mainly considers the energy dissipation of communication modules of nodes. The energy consumption of transmitting a packet of size b from $n_{i}$ to $n_{j}$ is expressed as

(1) $$E_{\mathrm{tx}}(i,j,b)=\left\{\begin{array}{cc} (b\cdot E_{\mathrm{elec}})+(b\cdot \zeta_{\mathrm{fs}}\cdot d_{i,j}^{2}), & d_{i,j}<d_{\mathrm{thr}}\\ (b\cdot E_{\mathrm{elec}})+(b\cdot \zeta_{\mathrm{amp}}\cdot d_{i,j}^{4}), & d_{i,j}\geq d_{\mathrm{thr}} \end{array}\right.$$

Similarly, the energy consumption of receiving a *b*(bits) packet by $n_{j}$ is formulated as

(2) $$E_{\mathrm{rx}}(j,b)=b\cdot E_{\mathrm{elec}}$$

where $E_{\mathrm{elec}}$ represents the circuit energy consumption consumed by the sensor to send or receive each bit of data transmission, $\varepsilon_{\mathrm{fs}}$ and $\varepsilon_{\mathrm{amp}}$ are the expended amplification energy in free space and multipath fading space, respectively, while $d_{\mathrm{thr}}=\sqrt{\zeta_{\mathrm{fs}}/\zeta_{\mathrm{amp}}}$ is the threshold distance in meters, and $d_{i,j}$ denotes the Euclidean distance from sender $n_{i}$ to receiver $n_{j}$.

### 3.3. Problem Description

As shown in Figure 2, before forwarding the packet to the next hop routing node, the sender needs to construct a CFS within its communication range and copy the packet to all nodes in this set. However, if too many forwarders are selected, the number of packet replicas in the network will increase, resulting in higher communication energy consumption for the nodes. The surviving nodes will quickly die due to energy depletion, thereby exacerbating the intermittent connectivity problem of the post-disaster network. On the contrary, if the number of forwarders is too small, the packet delivery rate of the post-disaster network cannot be guaranteed, resulting in lower reliability of the post-disaster data transmission. Therefore, the size of the constructed CFS will affect the transmission performance of the entire post-disaster network. In the multi-hop routing network of Mine Internet of Things (MIoT), the post-disaster data transmission in the planar accident area has a directional characteristic towards the sink. In order to reduce the transmission energy consumption of residual nodes and improve the success rate of packet forwarding, the CFS should be constructed by selecting nodes with high residual energy, more neighboring nodes, and that are closer to the sink. After the CFS is determined, it is necessary to further optimize the packet forwarding strategy and select an optimal forwarder from the CFS as the relay node for the current sender. Since the energy of the surviving nodes in the accident area is limited and cannot be replenished after the disaster, energy conservation is a main consideration in post-disaster data transmission. In order to reduce the transmission energy consumption generated by forwarding packets, candidate nodes that are farther away from the sink and have lower residual energy should be avoided by selecting the correct relay nodes. In addition, in the accident rescue scenario of the fully mechanized mining face, surviving nodes will be damaged by the environment factor in the accident area and fail at any time, leading to an increase in the probability of invalid nodes being selected as relay nodes. During post-disaster data transmission, when invalid nodes are selected as relay nodes, data packets will be discarded by the nodes, which not only increases the network energy consumption but also reduces data transmission efficiency. In Figure 2, there are no available relay nodes within the forward communication range towards the sink for the invalid node. Therefore, it is necessary to find new recovery relay nodes on the reverse transmission path towards the sink and continue to forward packets.

## 4. Proposed DEOR Algorithm

To solve the routing problem described in Section 3.3, a Directional-area-forwarding-based Energy-efficient Opportunistic Routing (DEOR) for the post-disaster MIoT network is proposed, aiming to improve the energy efficiency and the data transmission reliability of the post-disaster MIoT network in the planar accident mine. The proposed DEOR algorithm mainly consists of three parts: candidate forwarding set construction, relay node selection, and void routing node recovery. Firstly, a directional-area-forwarding-based candidate forwarding set construction strategy is designed. In the initialization phase, according to the deployment density and communication radius of nodes in the accident roadway, a forwarding zone (FZ) is designed for each node to route packets toward the sink. Then, the CFS is constructed by the nodes within the FZ that satisfy the energy constraint and the neighboring node degree constraint. Subsequently, a relay node selection method based on routing quality evaluation is proposed. In the data transmission phase, we take multiple attributes of nodes into account, such as direction angle, transmission distance, and residual energy. All nodes in the CFS are prioritized based on the routing quality evaluation, and the node with the highest priority is selected as the relay to forward packets. Finally, a recovery mechanism for void nodes is designed. When packets encounter the routing void during forwarding, a recovery mechanism is triggered. By employing the modified routing quality evaluation function, packets can bypass the void region and select available relay nodes to continue forwarding. Details are described below.

### 4.1. Construction of Candidate Forwarding Set

In this section, we mainly provide a detailed presentation of the construction of the candidate forwarding set (CFS). OR utilizes multiple neighbors of the sending node to simultaneously receive and forward packets, thus improving the packet forwarding efficiency. Therefore, the construction criterion of the CFS is particularly important in opportunity routing design.

**Figure 3:**
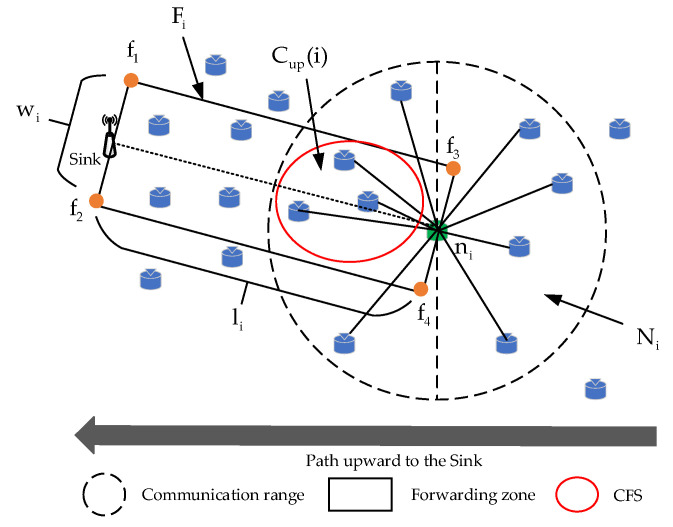
Schematic diagram of CFS selection of node *n_i_*.

In general, the size of the CFS can affect the post-disaster network performance. The larger the CFS, the more packet copies are generated and the higher the packet transfer success rate; however, the waiting time of the sender will also be longer, leading to higher energy consumption and end-to-end latency. On the contrary, the smaller the CFS, the sparser the network topology, and the lower the packet transfer success rate, resulting in unreliable data transmission after the disaster. To address the above issue, this paper proposes a directional-area-forwarding-based candidate forwarding set construction strategy. Here, we take the packet forwarding process from the source node $n_{i}$ to the sink in the post-disaster network as an example to build a schematic diagram of the CFS selection of node *n_i_*, as shown in Figure 3.

Figure 3 shows the selection of the CFS in the proposed DEOR algorithm. It can be seen that when the accident area is certain, the size of the CFS is related to the network density D. The higher the network density, the greater the probability of packets being overheard and duplicated. In addition, the direction of data transmission in the accident mine is upward to the sink. In order to reduce transmission energy consumption, nodes closer to the sink should be selected as much as possible when constructing the CFS. Therefore, to restrict the number of forwarders and reduce the energy consumption, we defined a Forwarding Zone (FZ) for each node $n_{i}$, denoted by $F_{i}$, so that the packets will be routed upwards to the sink within the $F_{i}$. The size of the FZ is determined by the network density D; larger network density means a smaller FZ and fewer forwarders, and vice versa, such that the number of forwarders can be dynamically adjusted. Here, we denote the network density D as

(3) $$D=\frac{1}{2}\left(\frac{4\pi |N|r^{2}}{A}+\frac{\sum_{i=0}^{\left|N\right|}m_{i}}{\left|N\right|}\right)=\frac{1}{2A|N|}\left(4\pi |N{|}^{2}r^{2}+A\sum_{i=0}^{\left|N\right|}m_{i}\right)$$

where the first term reflects the deployment density in the target field, and the second term reflects the degree of connections between the sender and other nodes. Note that, $\left(\pi |N|r^{2}\right)$ is the sum area of |N| nodes, |N| is the size of post-disaster network, r is the communication radius of nodes, A is the area of target field, and $m_{i}$ is the number of neighbors of $n_{i}$.

In Figure 3, it can be seen that FZ is a rectangular shape with a size of $l_{i}\times w_{i}$ defined by four points $\left(f_{1},f_{2},f_{3},f_{4}\right)$, where the length of the FZ is the Euclidean distance from the node to the sink, i.e., $l_{i}=d_{i,\mathrm{sink}}$. Obviously, the width of the FZ $w_{i}$ is related to the communication range r and the network density D. According to the model studied in [37], the maximum width of the FZ satisfies $w_{i,\max}\leq 4r$. Consequently, the $w_{i}$ is expressed as

(4) $$w_{i}=\frac{4r}{\sqrt{D}}=\frac{4r\sqrt{2A|N|}}{\sqrt{4\pi |N{|}^{2}r^{2}+A\sum_{i=0}^{\left|N\right|}m_{i}}}$$

According to Formula (4), there is a positive correlation between the width $w_{i}$ of the FZ and the communication radius *r* of the sender $n_{i}$, and a negative correlation between the width $w_{i}$ and the network density D. When the communication radius r of node $n_{i}$ is fixed, the larger the network density D is, the smaller the width $w_{i}$ of the sender $n_{i}$. This is because a larger network density D means more candidate forwarding nodes of $n_{i}$, leading to an increase in packet replicas in the post-disaster network. To reduce the number of neighbors participating in packet forwarding, the width of the FZ $w_{i}$ should be reduced. Conversely, the smaller the network density D, the larger the width $w_{i}$ of the sender $n_{i}$. This is because the smaller the network density D, the fewer candidate forwarding nodes of $n_{i}$ there are, leading to a lower packet success delivery rate. Therefore, under the condition of a fixed sensing region, the width of FZ $w_{i}$ should be increased to improve the probability of packet forwarding by relay routing nodes.

The location of the sink is denoted by $\left(x_{\mathrm{sink}},y_{\mathrm{sink}}\right)$. In the network initialization phase, each node $n_{i}$ computes the location of the four points of $F_{i}$ using Formula (5). These four points $\left(f_{1},f_{2},f_{3},f_{4}\right)$ are attached to the header of packets. Obviously, the number of forwarders can be limited according to the FZ.

(5) $$\begin{array}{l} f_{1}=\left(x_{\mathrm{sink}}+\frac{w_{i}(y_{\mathrm{sink}}-y_{i})}{2d_{i,\mathrm{sink}}},y_{\mathrm{sink}}+\frac{w_{i}(x_{i}-x_{\mathrm{sink}})}{2d_{i,\mathrm{sink}}}\right)\\ f_{2}=\left(x_{\mathrm{sink}}-\frac{w_{i}(y_{\mathrm{sink}}-y_{i})}{2d_{i,\mathrm{sink}}},y_{\mathrm{sink}}-\frac{w_{i}(x_{i}-x_{\mathrm{sink}})}{2d_{i,\mathrm{sink}}}\right)\\ f_{3}=\left(x_{i}+\frac{w_{i}(y_{\mathrm{sink}}-y_{i})}{2d_{i,\mathrm{sink}}},y_{i}+\frac{w_{i}(x_{i}-x_{\mathrm{sink}})}{2d_{i,\mathrm{sink}}}\right)\\ f_{4}=\left(x_{i}-\frac{w_{i}(y_{\mathrm{sink}}-y_{i})}{2d_{i,\mathrm{sink}}},y_{i}-\frac{w_{i}(x_{i}-x_{\mathrm{sink}})}{2d_{i,\mathrm{sink}}}\right) \end{array}$$

We define the neighbor set of $n_{i}$ within the forwarding zone as $F_{i}$. In Figure 3, the candidate zone (CZ) of each node $n_{i}$ is defined as the intersection area between the forwarding zone $F_{i}$ and the communication range of $n_{i}$. Furthermore, the set of nodes within the CZ is described as

(6) $$Z_{i}=\{n_{j}|n_{j}\in (F_{i}\cap N_{i})\}$$

In practice, due to the harsh underground mine environment after the disaster, there still exists the problem of node failure at any time. Therefore, in order to avoid encountering void routing during packet forwarding, candidate nodes with more neighbor nodes should be selected so that the packet transmission success rate can be improved. In addition, candidate nodes with relatively high residual energy should be selected to balance the node load and extend the network lifetime. Consequently, the node $n_{j}$ in the CFS of node $n_{i}$ should satisfy Formulas (7) and (8).

(7) $$N(m_{i})=\left\{\{n_{j},n_{v}\}\in N_{i}|m_{j}\geq \frac{\sum_{v}m_{v}}{m_{i}}\right\}$$

(8) $$N(\varepsilon_{i})=\left\{\{n_{j},n_{v}\}\in N_{i}|\varepsilon_{j}\geq \frac{1}{2}\frac{\sum_{v}\varepsilon_{v}}{m_{i}}\right\}$$

where $m_{j}$ and $m_{v}$ represent the number of neighbor nodes of $n_{j}$ and $n_{v}$, respectively, while $\varepsilon_{j}$ and $\varepsilon_{v}$ represent the residual energy of $n_{j}$ and $n_{v}$, respectively.

As a result, the nodes satisfying Formulas (6)–(8) constitute the candidate forwarding set $C_{\mathrm{up}}(i)$ of $n_{i}$ as follows:

(9) $$C_{\mathrm{up}}(i)=Z_{i}\cap N(m_{i})\cap N(\varepsilon_{i})$$

In this article, the constructed candidate forwarding set $C_{\mathrm{up}}(i)$ restricts the number of forwarders, which contributes to reducing the waiting time of the sender. Moreover, the nodes in $C_{\mathrm{up}}(i)$ have the characteristics of high energy and more neighboring nodes, which is conducive to improving the network lifetime and data transmission reliability of post-disaster MIoT. The pseudocode of the selection of the candidate forwarding set is shown in Algorithm 1.
**Algorithm 1:** Construct the Candidate Forwarding Set**Input:** $C_{\mathrm{up}}(i)=\emptyset$ **Output:** The candidate forwarding set $C_{\mathrm{up}}(i)$ 1: **for** each node $n_{i}\in N$ **do** 2:        Define the Forwarding Zone $F_{i}$ using Equation (5) 3:        Get the subset $Z_{i}$ using Equation (6) 4: **end for** 5: **for** each node $n_{j}\in N_{i}$ do 6:        Get the subset $N(m_{i})$ using Equation (7) 7:        Get the subset $N(\varepsilon_{i})$ using Equation (8) 8:    **if**
$n_{j}\in Z_{i}$$\;\&\&\;n_{j}\in N(m_{i})$$\&\&n_{j}\in N(\varepsilon_{i})$ 9:        **then** add $n_{v}\to C_{\mathrm{up}}(i)$
10:     **end if** 11: **end for** 12: **if**
$C_{\mathrm{up}}(i)!=\emptyset$ 13:       **then**
$C(i)=C_{\mathrm{up}}(i)$ 14:       switch to **Algorithm 3** 15: **else**
16:       switch to **Algorithm 2** 17: **end if**

### 4.2. Selection of Relay Node

**Figure 4:**
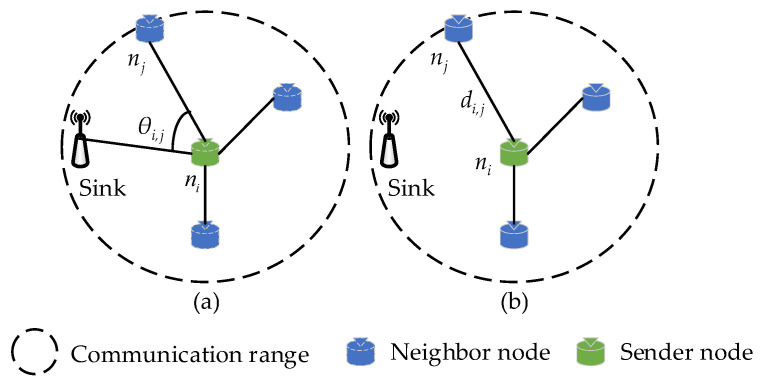
(**a**) The direction angle $\theta_{i,j}$ from $n_{i}$ to $n_{j}$ with respect to the location of the sink. (**b**) The transmission distance $d_{i,j}$ from $n_{i}$ to $n_{j}$.

In this section, the proposed relay node selection method based on routing quality is described in detail. In opportunistic routing, the relay node is the ultimate node responsible for packet forwarding, so the selection of relay nodes will directly affect the performance of data transmission in the post-disaster Mine Internet of Things (MIoT). After the candidate forwarding set is determined, we need to further optimize the forwarding strategy with the goal of selecting the optimal forwarder as the next-hop relay node. Based on the system model shown in Figure 2, aiming to reduce the energy consumption for data transmission in the post-disaster MIoT, an energy-efficient routing path should be selected from the source to the sink [38]. Here, we design a routing quality evaluation function in the proposed DEOR algorithm for forwarders that considers three factors, including direction angle, transmission distance, and residual energy of nodes. The smaller the directional angle attribute value of the current forwarder, the closer the forwarder is to the sink and the lower the transmission energy consumption of sensor nodes. The larger the relative distance attribute value between the sender and forwarder, the farther the transmission distance of the current forwarder is within the same communication range, which shortens the total routing path for packet forwarding and thus reduces the transmission energy consumption of the post-disaster network. In this article, the schematic diagram of the directional angle and transmission distance between the sender $n_{i}$ and forwarder $n_{j}$ is shown in Figure 4. Then, nodes in the CFS are prioritized based on the routing quality value. After the forwarder’s coordination, the node with the highest priority is selected as the relay to forward packets, and other nodes in the CFS will drop packets after listening for a successful transmission message.

As shown in Figure 4a, the directional angle attribute of the node is considered in this paper to give higher priority to forwarders closer to the sink. The direction angle $\theta_{i,j}$ between the sender $n_{i}$ and the neighbor $n_{j}$ towards the sink is expressed by Formula (10), where $\vec{a}=(x_{j}-x_{i},y_{j}-y_{i})$ and $\vec{c}=(x_{\mathrm{sink}}-x_{i},y_{\mathrm{sink}}-y_{i})$. Figure 4a shows that the smaller the direction angle $\theta_{i,j}$ between the sender $n_{i}$ and its neighbor $n_{j}$ is, the closer the neighbor $n_{j}$ is to the sink, where it can provide lower energy consumption for packet forwarding. To avoid the situation where $\theta_{i,j}=0$, the $\theta_{i,j}$ is normalized into ${\bar{\theta }}_{i,j}$ by Formula (11), where $1\leq {\bar{\theta }}_{i,j}\leq 2$. Then, the distribution of the direction angle ${\tilde{\theta }}_{i,j}$ is obtained by using the mass function expressed as Formula (12).

(10) $$\theta_{i,j}=\arccos\frac{\vec{a}\cdot \vec{c}}{\left||\vec{a}||\cdot ||\vec{c}|\right|}$$

(11) $${\bar{\theta }}_{i,j}=\frac{3-\theta_{i,j}}{2}$$

(12) $${\tilde{\theta }}_{i,j}=\frac{e^{-{\left({\bar{\theta }}_{i,j}\right)}^{\alpha_{\theta }}}}{\sum_{k=0}^{c_{i}}e^{-{\left({\bar{\theta }}_{i,k}\right)}^{\alpha_{\theta }}}}$$

where $c_{i}$ is the number of nodes in C(i), $\left\{n_{j},n_{k}\}\in C(i\right)$, and $\alpha_{\theta }(\alpha_{\theta }\geq 0)$ is the control parameter of the direction angle factor. Note that a larger $\alpha_{\theta }$ indicates the greater probability distribution for forwarders that are closer to the sink to be selected as relay nodes.

As shown in Figure 4b, the transmission distance attribute of nodes is considered in our work to give higher priority to forwarders that are further away from the sender. This is because when the communication radius r of nodes is fixed, the larger the distance between the sender $n_{i}$ and its neighbor $n_{j}$ is, the shorter the routing path toward the sink becomes, thereby reducing the energy consumption for routing packets. The transmission distance $d_{i,j}$ between $n_{i}$ and $n_{j}$ is represented by Formula (13). The variable $d_{i,j}$ is normalized to ${\bar{d}}_{i,j}$ by Formula (14), where ${\bar{d}}_{i,j}\in [1,2]$. Then, the distribution of transmission distance ${\tilde{d}}_{i,j}$ is obtained by using the mass function expressed as Formula (15).

(13) $$d_{i,j}=\sqrt{{\left(x_{i}-x_{j}\right)}^{2}+{\left(y_{i}-y_{j}\right)}^{2}}$$

(14) $${\bar{d}}_{i,j}=\frac{d_{i,j}+r}{r}$$

(15) $${\tilde{d}}_{i,j}=\frac{e^{{\left({\bar{d}}_{i,j}\right)}^{\alpha_{d}}}}{\sum_{k=0}^{c_{i}}e^{{\left({\bar{d}}_{i,k}\right)}^{\alpha_{d}}}}$$

where $\alpha_{d}(\alpha_{d}\geq 0)$ is the control parameter of the transmission distance factor. Note that a larger $\alpha_{d}$ indicates the greater probability distribution for forwarders that are closer to the sink to be selected as relay nodes.

Furthermore, some network nodes can deplete energy earlier than other nodes due to undertaking more packet forwarding in data transmission. In order to balance the load of network nodes, the energy attribute of nodes is also considered in our work to give higher priority to forwarders with greater residual energy. For a forwarder $n_{j}$ of the sender $n_{i}$, the residual energy $\varepsilon_{i,j}$ is normalized to ${\bar{\varepsilon }}_{i,j}$ by Formula (16). Then, the distribution of remaining energy ${\tilde{\varepsilon }}_{i,j}$ is obtained by using the mass function expressed as Formula (17).

(16) $${\bar{\varepsilon }}_{i,j}=\frac{\varepsilon_{i,j}+e_{\mathrm{ini}}}{e_{\mathrm{ini}}}$$

(17) $${\tilde{\varepsilon }}_{i,j}=\frac{e^{{\left({\bar{\varepsilon }}_{i,j}\right)}^{\alpha_{\varepsilon }}}}{\sum_{k=0}^{c_{i}}e^{{\left({\bar{\varepsilon }}_{i,k}\right)}^{\alpha_{\varepsilon }}}}$$

where $e_{\mathrm{ini}}$ is the initial energy of $n_{j}$ and $\alpha_{\varepsilon }(\alpha_{\varepsilon }\geq 0)$ is the control parameter of the energy factor. Note that a larger $\alpha_{\varepsilon }$ indicates the greater probability distribution for forwarders that have greater remaining energy to be selected as relay nodes.

Based on the above analysis, we define the routing quality of $n_{i}$’s forwarder $n_{j}$ as the product of the directional angle factor ${\tilde{\theta }}_{i,j}$, the transmission distance factor ${\tilde{d}}_{i,j}$, and the residual energy factor ${\tilde{\varepsilon }}_{i,j}$, which is expressed as ${\tilde{Q}}_{i,j}$ in Formula (18). Then, it is normalized to ${\bar{Q}}_{i,j}$ by Formula (19).

(18) $${\tilde{Q}}_{i,j}={\tilde{\theta }}_{i,j}\times {\tilde{d}}_{i,j}\times {\tilde{\varepsilon }}_{i,j}$$

(19) $${\bar{Q}}_{i,j}=\frac{{\tilde{Q}}_{i,j}}{\sum_{k=0}^{c_{i}}{\tilde{Q}}_{i,k}}=\frac{e^{\left({\left({\bar{\varepsilon }}_{i,j}\right)}^{\alpha_{\varepsilon }}-{\left({\bar{\theta }}_{i,j}\right)}^{\alpha_{\theta }}+{\left({\bar{d}}_{i,j}\right)}^{\alpha_{d}}\right)}}{\sum_{k=0}^{c_{i}}e^{\left({\left({\bar{\varepsilon }}_{i,k}\right)}^{\alpha_{\varepsilon }}-{\left({\bar{\theta }}_{i,k}\right)}^{\alpha_{\theta }}+{\left({\bar{d}}_{i,k}\right)}^{\alpha_{d}}\right)}}$$

According to Formula (19), we can deduce that the forwarder with higher routing quality ${\bar{Q}}_{i,j}$ has a higher chance of being selected as a relay node. Note that these three factors are controlled by three control parameters $\alpha_{\theta }$, $\alpha_{d}$, and $\alpha_{\varepsilon }$, respectively, such that increasing the value of any control parameter will enhance the impact of the corresponding indicator. Normally, the control parameters are set to $\alpha_{\theta }=\alpha_{d}=\alpha_{\varepsilon }=1$.

**Figure 5:**
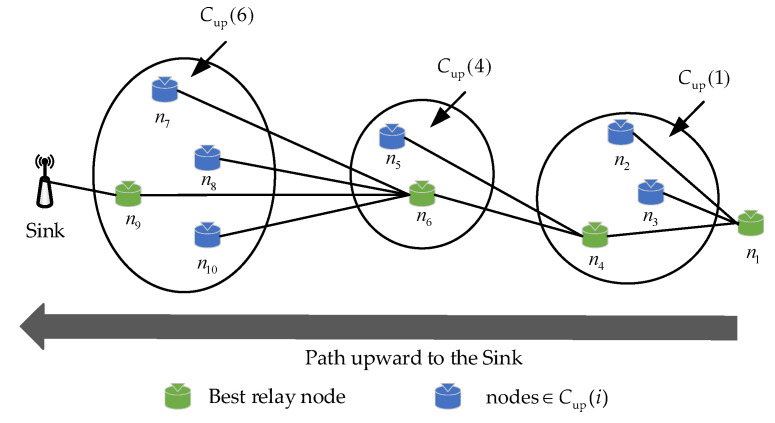
Schematic diagram of relay node selection from source *n*_1_ to the sink.

Once the CFS of the sender is determined, the number of packet replicas needs to be limited by the collaboration of candidate forwarders, ensuring that only one forwarder is selected as the relay node to forward packets. In this article, the proposed relay node selection method based on routing quality evaluation determines the optimal relay node for packet forwarding, so that an energy-efficient routing path between the sender and the sink can be achieved. By utilizing the local metrics of forwarders to make routing decisions, DEOR reduces routing overhead and extends network lifetime. The schematic diagram of relay node selection is shown in Figure 5.

According to the routing quality ${\bar{Q}}_{i,j}$ value, we sort the forwarders of $n_{1}$ in descending order, denoted as $C({\bar{Q}}_{i})$. As we can see in Figure 5, for the current sender $n_{1}$ carrying packets, $C_{\mathrm{up}}(1)$ is the candidate forwarding sets of $n_{1}$, where $C_{\mathrm{up}}(1)=\{n_{2},n_{3},n_{4}\}$. When $n_{1}$ needs to find the next-hop routing node, it will send a request message to all nodes in $C_{\mathrm{up}}(1)$. After receiving this message, these forwarders send a reply message about their own information to $n_{1}$. Then, according to Formulas (12), (15) and (17), $n_{1}$ calculates the three attribute values of nodes $n_{2}$, $n_{3}$, and $n_{4}$, i.e., $\left({\tilde{\theta }}_{1,2},{\tilde{d}}_{1,2},{\tilde{\varepsilon }}_{1,2}\right)$, $\left({\tilde{\theta }}_{1,3},{\tilde{d}}_{1,3},{\tilde{\varepsilon }}_{1,3}\right)$, and $\left({\tilde{\theta }}_{1,4},{\tilde{d}}_{1,4},{\tilde{\varepsilon }}_{1,4}\right)$. Next, $n_{1}$ calculates the routing quality values of three forwarders based on Formula (19), i.e., ${\bar{Q}}_{1,2}$, ${\bar{Q}}_{1,3}$, and ${\bar{Q}}_{1,4}$. Finally, the three routing quality values are compared. Assuming that ${\bar{Q}}_{1,4}>{\bar{Q}}_{1,3}>{\bar{Q}}_{1,2}$, the forwarders’ priority sorting set of the sender $n_{1}$ can be denoted as $C({\bar{Q}}_{1})=\{n_{4},n_{3},n_{2}\}$. The sender $n_{1}$ selects the candidate with the largest ${\bar{Q}}_{i,j}$ value as the next hop, i.e., ${\bar{Q}}_{1,4}$. The best relay node $n_{4}$ forwards the packets, and if the transmission is successful, the other nodes $n_{2}$ and $n_{3}$ discard the packet copies. Since $n_{4}$ is not the destination node, it becomes the new sender and continues to select the next hop through the above process until the packet is forwarded to the sink. Finally, a complete routing path is formed in the network, denoted as $\left(n_{1},\;n_{4},\;n_{6},\;n_{9},\;\mathrm{Sink}\right)$ in Figure 5. The pseudocode of the selection of relay nodes is shown in Algorithm 2.
**Algorithm 2:** Select the Best Relay Nodes$\mathrm{Input}:\;{\bar{Q}}_{i,j}=({\bar{Q}}_{i,1},{\bar{Q}}_{i,2},\ldots ,{\bar{Q}}_{i,c_{i}})$ Output: The ID of the best relay nodes 1: **for** each node $n_{j}\in C(i)$ do 2:       node $n_{j}$ receives the packets sent by node $n_{i}$ 3:       Get the ${\tilde{\theta }}_{i,j}$ using Equation (12) 4:       Get the ${\tilde{d}}_{i,j}$ using Equation (15) 5:       Get the ${\tilde{\varepsilon }}_{i,j}$ using Equation (17) 6:       Calculate ${\bar{Q}}_{i.j}$ using Equation (19) 7:       sort $\left({\bar{Q}}_{i,1},{\bar{Q}}_{i,2},\ldots ,{\bar{Q}}_{i,c_{i}}\right)$ in descending order to $C({\bar{Q}}_{i})$ 8: **end for** 9: select the node $n_{j}$ from the highest—$C({\bar{Q}}_{i})$ 10: **if**
$n_{j}$ forwards the packet successfully 11:    **then** other nodes in C(i) drop the packet 12: **else** 13:       set the node $n_{j}\;=\;n_{v}$ where $n_{j}$ has lower—$C({\bar{Q}}_{i})$ 14: **end if** 15: **until** the timer expired 16: **if** packet is not delivered to Sink 17:       **then**
$n_{\mathrm{sender}}=n_{j}$ 18:       switch to **Algorithm 1** 19: **end if**

### 4.3. Recovery Mechanism

**Figure 6:**
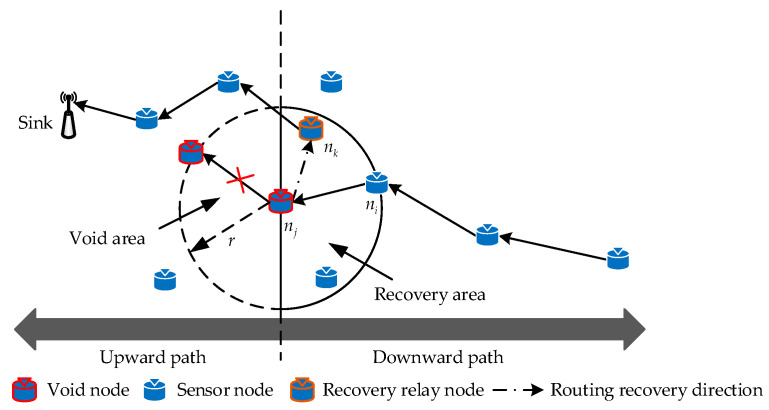
Schematic diagram of routing recovery mechanism from void node *n_j_* to relay node *n_k_*.

In this section, we describe in detail a recovery mechanism in the proposed DEOR algorithm. The collapse of loose coal in mines after disasters and the depletion of node energy are common phenomena that can increase the probability of void nodes being selected as relay nodes [39]. However, although the above methods reduce the probability of selecting void nodes, they cannot completely avoid the problem of void routing. According to Figure 2, we assume that the routing node $n_{j}$ of the current sender $n_{i}$ is an invalid node; the routing recovery process of void nodes in the post-disaster MIoT network is shown in Figure 6. By adopting the proposed recovery mechanism, the current void node $n_{j}$ can quickly find the optimal relay recovery node $n_{k}$ on the reverse routing path towards the sink, thereby bypassing the void area and improving the packet delivery rate of post-disaster network data transmission.

In Figure 6, if the relay node $n_{j}$ of the sender $n_{i}$ is a void node, no neighbors in the upward path to the sink can forward packets, i.e., the candidate forwarder set $C_{\mathrm{up}}(j)$ obtained according to Algorithm 1 is empty. Here, we denote the set of neighbor nodes of the void node $n_{j}$ as $N_{j}$, and its subset of neighbors $N(d_{j})$ in the downward path can be defined as

(20) $$N(d_{j})=\{n_{k}\in N_{j}\subseteq N\;|\;d_{k,\mathrm{sink}}-d_{j,\mathrm{sink}}\geq 0\}$$

where $n_{k}$ is the neighbor of void node $n_{j}$ and $d_{j,\mathrm{sink}}=\sqrt{{\left(x_{j}-x_{\mathrm{sink}}\right)}^{2}+{\left(y_{j}-y_{\mathrm{sink}}\right)}^{2}}$ represents the Euclidean distance from $n_{j}$ to the sink.

Similar to Formula (8), in order to ensure load balancing and energy conservation in the post-disaster network, nodes with higher residual energy should be selected. Hence, the subset $N(\varepsilon_{j})$ of *n_j_*’s neighbors that satisfy energy constraints is denoted as

(21) $$N(\varepsilon_{j})=\left\{\{n_{k},n_{l}\}\in N_{j}\subseteq N|\varepsilon_{k}\geq \frac{1}{2}\frac{\sum_{l}\varepsilon_{l}}{\left(m_{j}\right)}\right\}$$

Combining Formula (20) and Formula (21), the candidate recovery node set $C_{\mathrm{down}}(j)$ of void node $n_{j}$ can be defined as

(22) $$C_{\mathrm{down}}(j)=N(d_{j})\cap N(\varepsilon_{j})$$

Unlike the routing method described in Section 4.2, when an invalid node $n_{j}$ selects a recovery relay node in a downward path to the sink, it will consume more energy to forward packets to nodes at greater distances. Therefore, the neighbor node with a smaller transmission distance difference from the void node should be selected as the recovery relay node. Consequently, in the recovery mechanism, Formulas (15) and (19) should be rewritten as

(23) $${\tilde{d}}_{j,k}=\frac{e^{-{\left({\bar{d}}_{j,k}\right)}^{\alpha_{d}}}}{\sum_{k=0}^{c_{j}}e^{-{\left({\bar{d}}_{j,k}\right)}^{\alpha_{d}}}}$$

(24) $${\bar{Q}}_{j,k}=\frac{{\tilde{Q}}_{j,k}}{\sum_{l=0}^{c_{j}}{\tilde{Q}}_{j,l}}=\frac{e^{\left({\left({\bar{\varepsilon }}_{j,k}\right)}^{\alpha_{\varepsilon }}-{\left({\bar{\theta }}_{j,k}\right)}^{\alpha_{\theta }}-{\left({\bar{d}}_{j,k}\right)}^{\alpha_{d}}\right)}}{\sum_{k=0}^{c_{j}}e^{\left({\left({\bar{\varepsilon }}_{j,l}\right)}^{\alpha_{\varepsilon }}-{\left({\bar{\theta }}_{j,l}\right)}^{\alpha_{\theta }}-{\left({\bar{d}}_{j,l}\right)}^{\alpha_{d}}\right)}}$$

where $c_{j}$ is the number of candidate forwarders of void node $n_{j}$.

Once the void node forwards packets to a normal routing node, it exits the recovery mechanism and continues to route packets to the sink, as described in Section 4.1 and Section 4.2. In the recovery mechanism, nodes will record the ID of the previous hop node, and these nodes will not be repeatedly selected when selecting the recovery relay node, thus avoiding routing loops. By utilizing the updated routing quality assessment, void nodes can select appropriate recovery relay nodes downward in the planar accident mine, thereby effectively restoring the transmission path. The pseudocode of the recovery mechanism is shown in Algorithm 3.
**Algorithm 3:** Recovery Mechanism of Void Nodes**Input:** $N_{i}$ **Output:** The candidate recovery forwarding set $C_{\mathrm{down}}(i)$ 1: **for** each node $n_{j}\in N_{i}$ **do** 2:        Get the subset $N(d_{j})$ using Equation (20) 3:        Get the subset $N(\varepsilon_{j})$ using Equation (21) 4:        **if**
$n_{j}\in N(d_{j})\&\&\;n_{j}\in N(\varepsilon_{j})$ 5:            **then** add $n_{j}\to C_{\mathrm{down}}(i)$ 6:          $C(i)=C_{\mathrm{down}}(i)$ 7:        **end if** 8: **end for** 9: switch to **Algorithm 2**

### 4.4. Analysis and Flowchart of DEOR

According to the pseudocode of the three sub-algorithms, e.g., Algorithms 1–3, the DEOR algorithm mainly consists of a cycle in the calculation process, so the computational complexity of the proposed DEOR in this paper is O(N), where N is the number of nodes in the network. The energy consumption in DEOR entirely depends on how many nodes are in the sender’s forwarding zone. For the post-disaster MIoT network, this complexity is usually within the computing capacity of nodes, and the proposed DEOR is an energy-saving routing strategy. Therefore, the surviving nodes have the ability to execute the DEOR algorithm, which restricts the number of duplicate packets generated in the network and avoids the routing void problem during data transmission.

**Figure 7:**
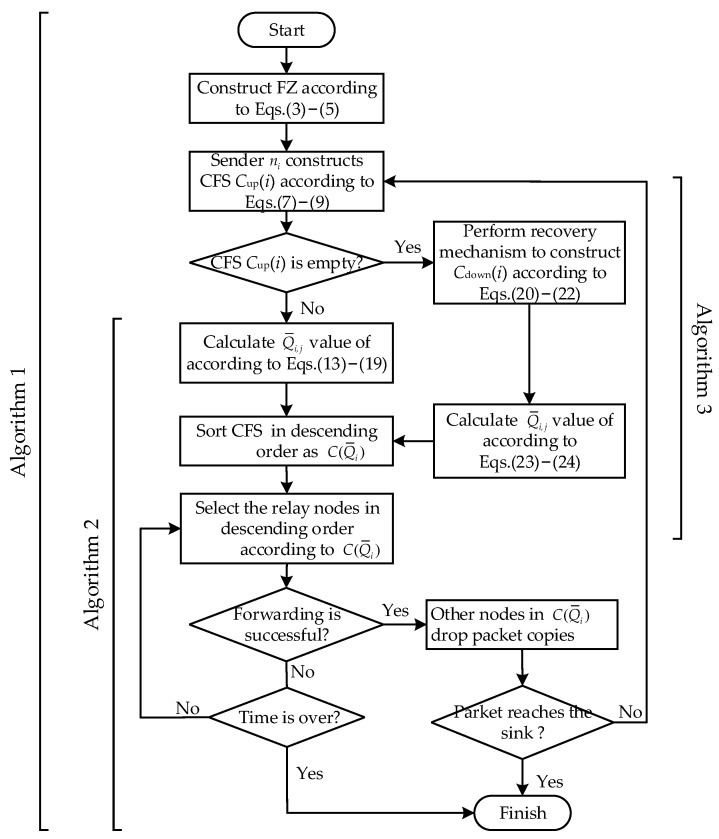
Flowchart of the proposed DEOR algorithm.

The proposed DEOR is an opportunistic routing algorithm that considers both global and local information of the network. Nodes make routing decisions based on network density and multiple attributes of neighboring nodes. The flowchart of DEOR is shown in Figure 7. Firstly, during the network initialization phase, each surviving node defines a forwarding zone (FZ) according to Formulas (3)–(5). It is assumed that the source node $n_{i}$ needs to send the perceived data information to the sink; that is, node $n_{i}$ is the current sender. Then, the sender $n_{i}$ constructs a candidate forwarding set (CFS) $C_{\mathrm{up}}(i)$ according to the forwarding zone constraint, energy constraint, and neighboring node degree constraint, i.e., Formulas (7)–(9). Next, the nodes within the $C_{\mathrm{up}}(i)$ calculate their routing quality ${\bar{Q}}_{i.j}$ values and are sorted in descending order to $C({\bar{Q}}_{i})$. In addition, if the set $C_{\mathrm{up}}(i)$ is empty, the recovery mechanism is activated. The sender $n_{i}$ reconstructs a new forwarder set $C_{\mathrm{down}}(i)$ according to Formulas (20)–(22). Then, the nodes within the $C_{\mathrm{down}}(i)$ calculate their routing quality ${\bar{Q}}_{i.j}$ values and are sorted in descending order to $C({\bar{Q}}_{i})$. The nodes in $C({\bar{Q}}_{i})$ are selected in sequence as relay nodes to forward the packets before the timer expires; otherwise, the data transmission fails. Finally, if any node in $C({\bar{Q}}_{i})$ successfully forwards the packet, the other nodes will discard the packet copies and loop the above process until the packet is routed to the sink.

## 5. Performance Evaluation

In this section, a MATLAB R2018b simulator is applied to evaluate the performance of the DEOR algorithm [40]. First, the simulation settings are introduced before evaluations. Then, DEOR is compared with Opportunistic Routing based on Residual energy (ORR) (ORR) [41], neighbor coverage-based OR (OBRN) [42], and energy-efficient OR (ENSOR) [43] in terms of energy consumption, average hop count, packet delivery rate, and network lifetime. ORR is a centralized opportunistic routing algorithm based on distance and residual energy, OBRN is a distributed opportunistic routing algorithm based on energy and load, and ECSOR is a distributed opportunistic routing algorithm optimized for the forwarder node set.

### 5.1. Simulation Settings

In this paper, we consider the post-disaster data transmission of MIoT in the planar accident area rescue scenarios, such as a fully mechanized coal face [44,45]. Thus, we deploy all nodes randomly in a sensing area of 300 m×50 m in the simulation. This deployment approach ensures that the simulation results in different scenarios are independent of each other. Suppose a sink node with unlimited energy is fixed at the edge of the sensing area and acts as a gateway for wireless communication with sensor nodes. Similar to most wireless network routing protocols, we use the Carrier Sense Multiple Access (CSMA) protocol as the underlying MAC protocol. If the channel is idle, the forwarding node can broadcast the data packet. Otherwise, it backs off, and the packet will be dropped after backing off for the maximum retransmission times. The maximum retransmission times of each node is set to 4, and the maximum error ratio of each link is set to 0.2. We assume that the network generates a data packet from a random sensor node to the sink every 0.1 s. The size of the data packet is set to 1024 bits. The parameter settings required for the simulation are shown in Table 3.

The following routing metrics are used to evaluate the performance of routing algorithms: (1) energy consumption (EC): EC is defined as the total energy consumption required to deliver data packets from source nodes to the sink. (2) Average hop count (AHC): AHC is defined as the average hop count on the routing path from the source to the sink for forwarding data packets. (3) Packet delivery ratio (PDR): PDR is defined as the ratio of the number of packets successfully received at the sink to the number of data packets generated by source nodes. (4) Network lifetime (NL): NL is defined as the time (in seconds) from the simulation starting moment to the moment that the first node completely depletes its energy.

### 5.2. Effect of Control Parameters

**Figure 8:**
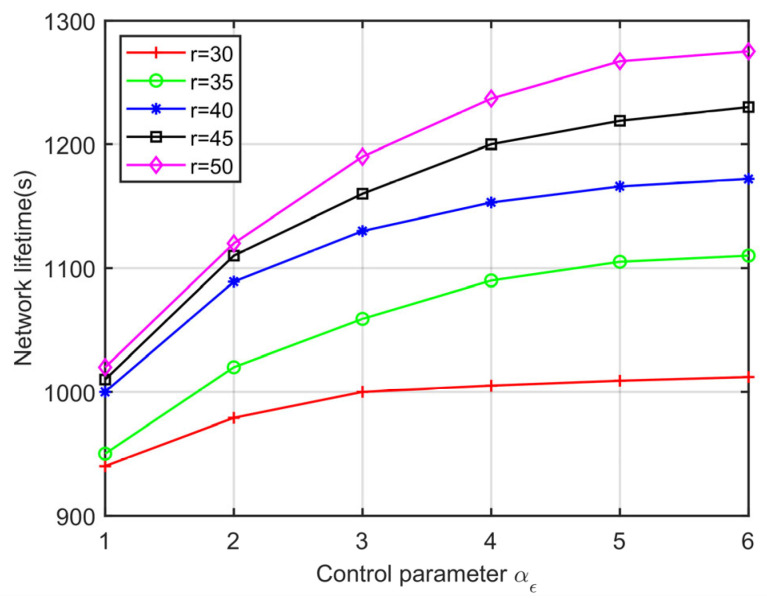
Network lifetime varying control parameters.

According to Formula (19), the routing quality is the multiplication product of three factors, i.e., ${\tilde{\theta }}_{i,j}$, ${\tilde{d}}_{i,j}$, and ${\tilde{\varepsilon }}_{i,j}$. Therefore, increasing the value of any control parameter will enhance the impact of the corresponding factors on routing performance. The control parameters $\left(\alpha_{\theta },\alpha_{d},\alpha_{\varepsilon }\right)$ can be adjusted to meet the requirements of the various applications for post-disaster MIoT. However, there may be a factor that is ineffective in routing in some cases. In order to select the optimal forwarders in the routing process, the setting of control parameter values should ensure that they have a certain effect on the three factors. In addition, in order to maximize the lifetime of the post-disaster network, the value of the energy control parameter $\alpha_{\varepsilon }$ should be larger than the other two parameters $\left(\alpha_{\theta },\alpha_{d}\right)$. In the simulation, the number of nodes is set to 200, the communication range of nodes varies from 30 m to 50 m, the two control parameters are set to $\alpha_{\theta }=\alpha_{d}=1$, and the value of $\alpha_{\varepsilon }$ varies from 1 to 6. Figure 8 shows the effect of the control parameter $\alpha_{\varepsilon }$ on the network lifetime. We can clearly see that when the communication range *r* is fixed, the network lifetime increases the larger $\alpha_{\varepsilon }$ is. When $\alpha_{\varepsilon }=5$, the growth of network lifetime tends to be stable. Therefore, the DEOR algorithm ensures good performance in terms of network lifespan for different control parameters’ $\alpha_{\varepsilon }$ values.

### 5.3. Performance for Varying Communication Ranges

**Figure 9:**
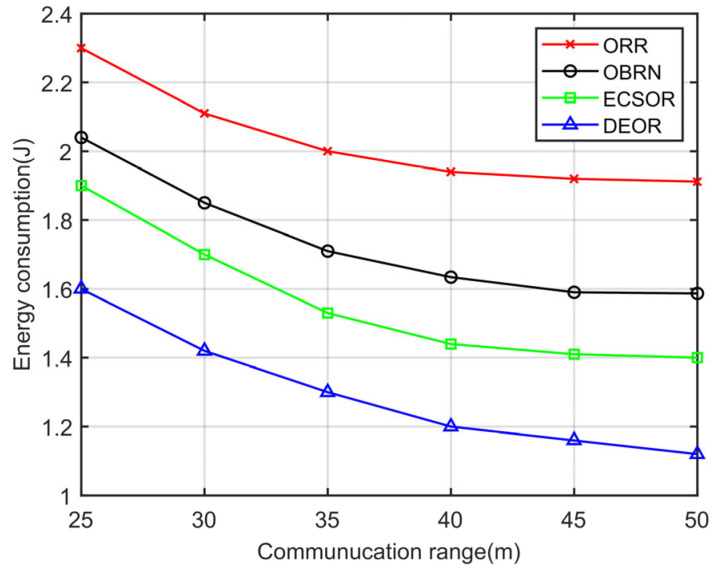
Energy consumption varying with communication range.

**Figure 10:**
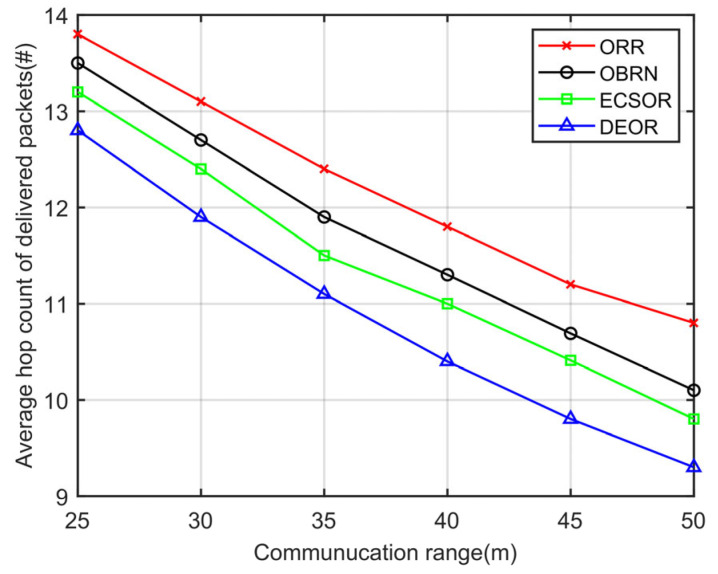
Average number of hops varying with communication range.

**Figure 11:**
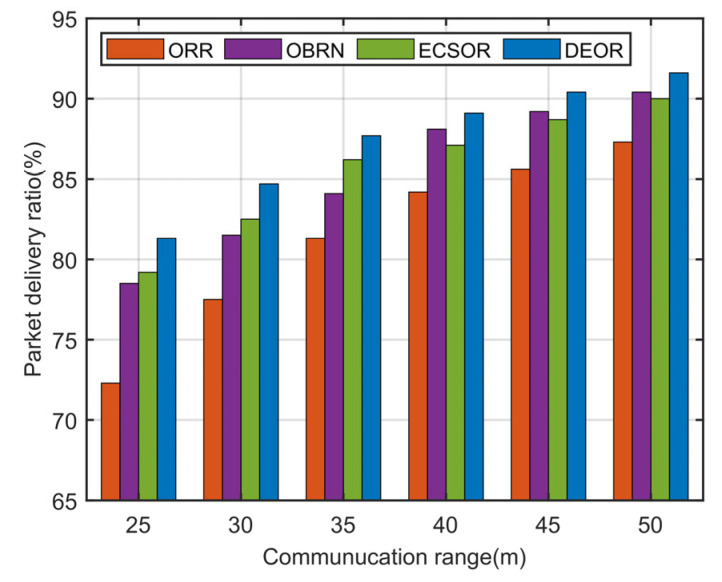
Packet delivery ratio varying with communication range.

**Figure 12:**
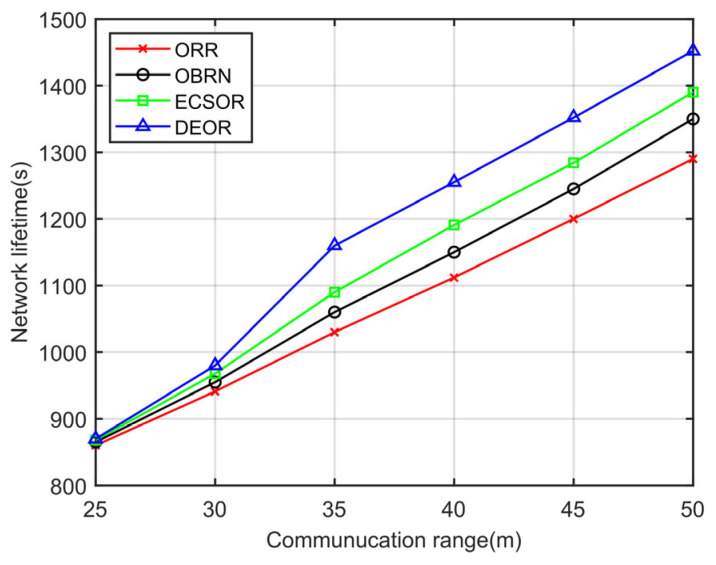
Network lifetime varying with communication range.

According to Formula (3), it can be seen that for a given sensing area, the network density D is determined by the number of nodes N and the communication range r. The more network nodes there are or the larger the communication range, the higher the network density. Accordingly, we evaluate the network performance of the DEOR algorithm by varying the communication ranges r and the number of nodes N. In the simulation, the control parameter is set to $\alpha_{\theta }=\alpha_{d}=\alpha_{\varepsilon }=1$, the number of nodes is set to 200, and the communication range r is varied from 30 m to 50 m. Other parameters are listed in Table 3. The results can be found in Figure 9, Figure 10, Figure 11 and Figure 12.

Figure 9 illustrates the influence of the communication range on the energy consumption. From Figure 9, we can see that the energy consumption of all four routing algorithms decreases with the increase in communication range r. This is because as the communication range r increases, the number of hops for packet forwarding to the sink will correspondingly decrease, which means that the number of nodes involved in forwarding decreases, and therefore the total energy consumption of the network is reduced. The network energy consumption in ORR is the highest because ORR is a centralized routing algorithm that utilizes a large number of control packets to transmit node information to the sink when calculating routing metrics, resulting in significant energy consumption. Compared with OBRN and ECSOR, DEOR has the least energy consumption. This is because, in DEOR, the number of forwarding nodes is restricted by the directional forwarding zone; see Formula (6). The sender will dynamically adjust the number of candidate forwarders based on the current communication range r, thereby reducing the number of packet replicas. In addition, based on Formula (17), DEOR balances the load of nodes close to the sink and improves the energy utilization efficiency of nodes. Therefore, DEOR has better energy efficiency than ORR, OBRN, and ECSOR.

Figure 10 illustrates the influence of the communication range on the average hop count of delivered packets. It can be clearly seen that as the communication range r increases, the number of routing hops decreases for all four algorithms. This is because the larger the communication range r, the greater the transmission distance of nodes, which means that the packet’s routing path from the source node to the sink is shortened, and therefore fewer nodes are involved in forwarding. Compared with ORR, OBRN, and ECSOR, DEOR has the least number of hops because the DEOR can adjust the density of candidate nodes in the target area based on the communication range r and thus dynamically select the appropriate number of forwarders. In addition, DEOR considers the relationship between link length and transmission range, thus ensuring a lower number of hops in the routing path.

Figure 11 illustrates the influence of the communication range on the packet delivery ratio. It is obvious from Figure 11 that DEOR is better than ORR, OBRN, and ECSOR, because the PDR of the network is related to the link quality and the number of forwarding nodes. The DEOR algorithm takes into account the effect of transmission distance $d_{i,j}$ on routing when selecting relay nodes, ensuring better path quality of packets throughout the entire routing process. Moreover, DEOR makes each node have multiple forwarders through a recovery mechanism for void routing, which means that when a sender encounters a void node, it can quickly find recovery relay nodes, which in turn improves the packet delivery rate. However, ORR, OBRN, and ECSOR only consider the energy and load factors to make routing decisions. When encountering void nodes, a large number of control packets need to be sent to update network information to calculate a new forwarder set, which leads to a higher probability of packet loss; see Formula (24). As a result, the DEOR algorithm outperforms the other three algorithms in terms of PDR.

Figure 12 illustrates the influence of the communication range on the network lifetime. As can be seen in Figure 12, the network lifetime of all four algorithms increases as the communication range r becomes larger. Compared to ORR, OBRN, and ECSOR, DEOR achieves the longest network lifetime, while ORR has the shortest network lifetime. In OBRN and ECSOR, the remaining energy of nodes around the sink is not considered when selecting forwarders. There exists routing nodes nearthe sink participating in excessive packet forwarding, leading to the hotspot problem. Thus, premature depletion of energy by critical routing nodes causes network connection interruption. However, in DEOR, only nodes in the upward path to the sink are eligible to forward packets, which effectively reduces the number of redundant packets and shortens the routing path. Meanwhile, DEOR balances the load of nodes through the transmission distance factor ${\tilde{d}}_{i,j}$ in routing decisions, thereby reducing energy consumption. As a result, DEOR has a longer network lifetime than ORR, OBRN, and ECSOR.

### 5.4. Performance for Varying Number of Nodes

**Figure 13:**
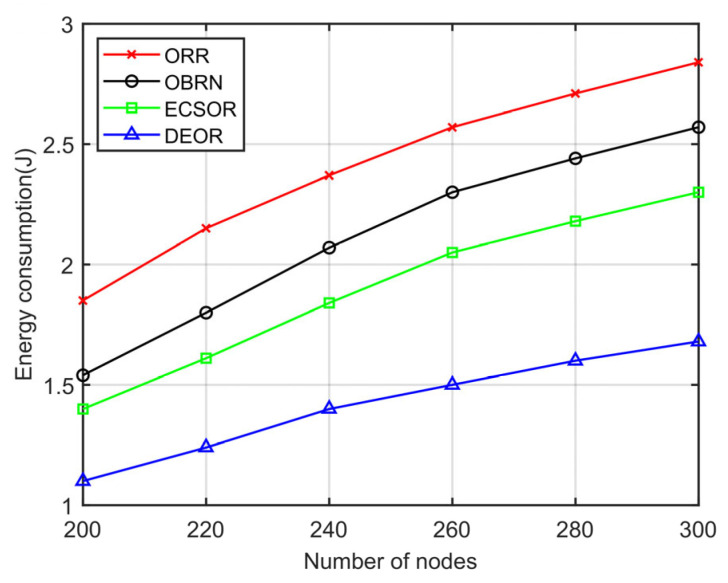
Energy consumption varying with number of nodes.

**Figure 14:**
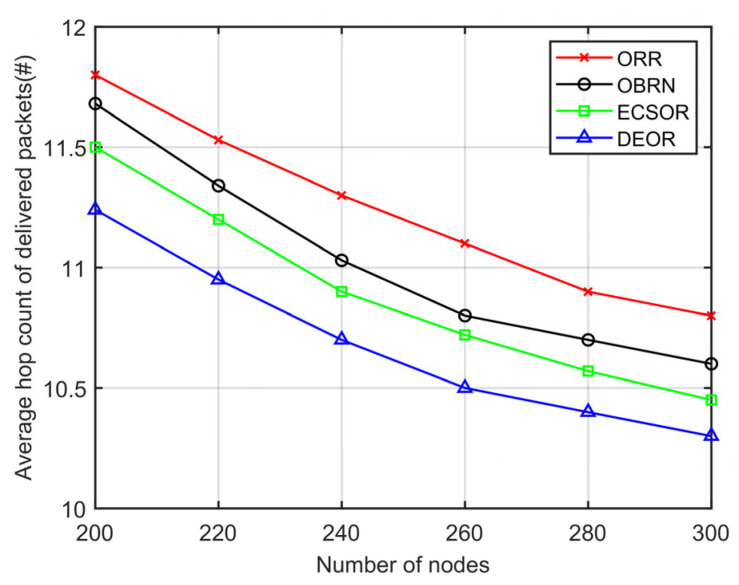
Average number of hops varying with number of nodes.

**Figure 15:**
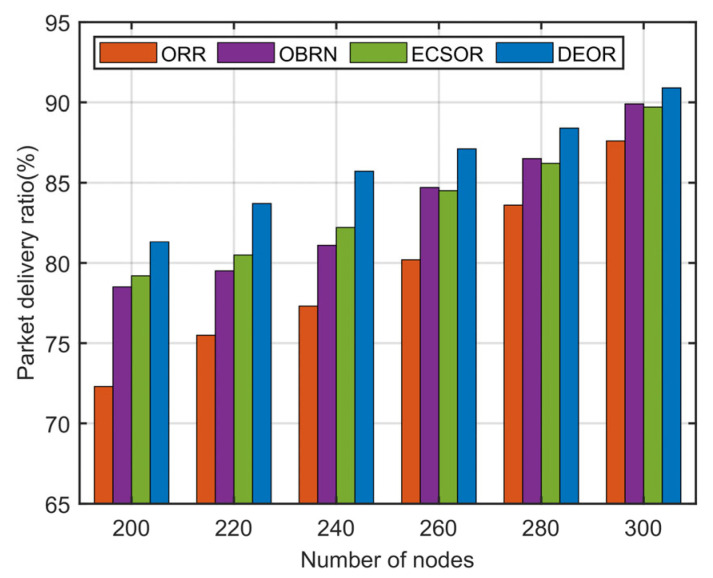
Packet delivery ratio varying with number of nodes.

**Figure 16:**
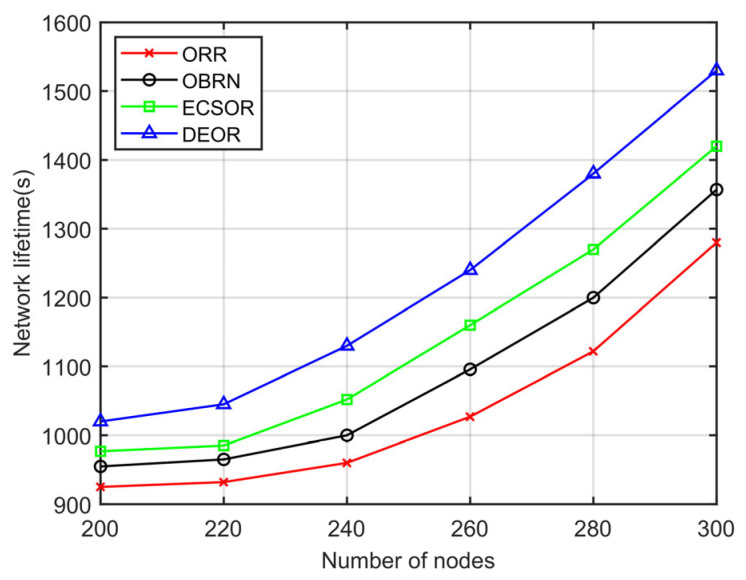
Network lifetime varying with number of nodes.

In this section, we evaluate the network performance of the DEOR algorithm by varying the number of nodes from 200 to 300. In the simulation, the control parameter is set to $\alpha_{\theta }=\alpha_{d}=1$, $\alpha_{\varepsilon }=5$, and the communication range is set to 40 m. Other parameters for this simulation are listed in Table 3. The results can be found in Figure 13, Figure 14, Figure 15 and Figure 16.

Figure 13 illustrates the influence of the number of nodes on the energy consumption. It is clearly seen that as the number of nodes becomes larger, the energy consumption of all four routing algorithms increases. This is because when the size of the monitoring area is fixed, more nodes in the network imply a higher network density, and then the number of nodes involved in forwarding increases. Excessive forwarding nodes will generate more redundant packets in the network, which in turn leads to higher energy consumption. DEOR has the smallest increase in energy consumption among the four algorithms because the number of forwarders in DEOR is controlled by the network density D. According to Formula (4), when the network density D increases, the width $w_{i}$ of the forwarding area will decrease, thereby limiting the number of nodes in the CFS. However, for the ORR, OBRN, and ECSOR algorithms, as the number of nodes in the network increases, the number of nodes participating in forwarding will also increase, generating more redundant packets and, therefore, leading to more energy consumption.

Figure 14 illustrates the influence of the number of nodes on the average hop count of delivered packets. It is observed that the average hop count in all four algorithms gradually decreases as the deployment density of nodes increases. This is because when the communication radius r of nodes is fixed, the number of nodes in the network is small, leading to a sparse local topology, and packets need to pass through a large number of relay nodes to enter the coverage range of the sink. Therefore, the average number of hops for packet forwarding is higher. On the contrary, when the number of nodes is higher, there are fewer void nodes, and packets are theoretically able to enter the coverage of the sink through the shortest path, resulting in a lower average number of hops. Compared with ORR, OBRN, and ECSOR, DEOR has the fewest number of hops. According to Formula (23), DEOR updates the transmission distance metric in the routing decision. DEOR selects optimal relay nodes closer to the void node by adopting a recovery mechanism, thereby shortening the length of the communication link.

Figure 15 illustrates the influence of the number of nodes on the packet delivery ratio. Obviously, the PDR of all four algorithms gradually increases as the number of nodes becomes larger. When the deployment density of nodes is higher, the probability of routing void areas decreases, and there are more available candidate nodes for packet forwarding. As a result, the probability of packet loss is reduced, and the packet delivery success rate is improved. ORR has the lowest PDR among the four routing algorithms. This is because only the residual energy of nodes is considered in ORR. If there are fewer nodes in the network, it is prone to a large amount of packet loss. Additionally, as shown in Figure 15, when the number of nodes approaches 300, the PDR of OBRN, ECSOR, and DEOR algorithms are close to the same, approximately 90%. However, when the number of nodes is less than 300, the DEOR algorithm is significantly better than OBRN and ECSOR. This is because when a sparse topology appears in the network, DEOR can increase the number of forwarders, thereby improving the reliability of successful packet forwarding.

Figure 16 illustrates the influence of the number of nodes on the network lifetime. From Figure 16, it can be seen that the network lifetime of the four routing algorithms increases as the number of network nodes increases. This is because DEOR designs a candidate forwarding zone (FZ) for all senders to make appropriate routing decisions. DEOR can limit the number of nodes involved in packet forwarding based on the current network density D, reducing the number of redundant packets generated in the network and thus reducing network energy consumption. Furthermore, DEOR utilizes a routing quality function to balance the traffic load of forwarding nodes, overcoming the problem of hot spots caused by critical routing nodes near the sink and thus improving the network lifetime.

## 6. Conclusions

In this paper, we propose a directional-area-forwarding-based energy-efficient opportunistic routing (shorted as DEOR) algorithm for the post-disaster MIoT network. Firstly, in order to restore post-disaster data transmission of MIoT in the planar accident area rescue scenarios, such as for a fully mechanized coal face, we design a multi-hop opportunity routing network architecture composed of one sink node and several survival sensor nodes. Then, according to the forwarding area constraint, energy constraint, and neighbor node degree constraint, DEOR constructs the candidate forwarding sets to restrict the number of duplicate packets and improve the energy utilization of nodes. Moreover, to meet multiple quality-of-service requirements of the post-disaster MIoT, a routing quality function is designed by considering the directional angle, transmission distance, and residual energy attributes of nodes. DEOR selects a relay node to forward packets based on the priority of the nodes in the CFS, which ensures the network load balancing. Finally, we propose a recovery mechanism aimed at bypassing the void area and forwarding the packets continuously, which can reduce retransmissions and improve network connectivity. The simulation results demonstrated the proposed DEOR algorithm achieves better performance compared to the ORR, OBRN, and ECSOR in terms of energy consumption, average hop count, packet delivery ratio, and network lifetime.

In our future work, we intend to investigate a more reasonable method to obtain the location information of sensor nodes for post-disaster mining scenarios. Moreover, how a realistic post-disaster mine roadway simulation experimental platform can be constructed is also a meaningful research topic.

## Figures and Tables

**Table 1:** Comparison of MIoT routing protocols.

Protocol	Scenario	Node Status	Deployment	Routing Metric	Features
RPAPC-MN [13]	Normal	Static and mobile	Partition	Area positive clustering	Reduces system energy consumption and extends network lifetime
DESR [14]	Normal	Static	Random	Transmission delay, packet loss rate, and energy consumption	Ensures QoS requirements
LBDA [15]	Normal	Static	Uniform	The forwarding data traffic and forwarding nodes	Balances node energy consumption and maximizes network lifecycle
SEC [16]	Post-disaster	Static	Random	Energy factor and connectivity factor	Extends network stability cycle and improves network stability
EAUC [17]	Post-disaster	Static	Random	Energy and distance factors	Balances cluster head energy consumption and improves data transmission
MVBN [18]	Post-disaster	Static	Random	The centrality of intermediate numbers, node compactness, and residual energy	Optimizes network remaining energy, number of dominant nodes, and node coverage
NHCRA-O [19]	Post-disaster	Static and mobile	Random	Residual energy factor, node connectivity, and directional medium	Improves node matching efficiency and network coverage efficiency
RIAC [20]	Post-disaster	Static	Random	Residual energy, distance, and trust factor	Reduces inter-cluster transmission energy consumption

**Table 2:** The notations used in this paper.

Notation	Meaning
N	The network. $n_{i}\in N$ is a sensor node.
$N_{i}$	The neighbor set of node $n_{i}$.
$m_{i}$	The size of $N_{i}$$,\;m_{i}=|N_{i}|$.
$n_{s}$	The source node.
$n_{\mathrm{sink}}$	The sink node.
$e_{\mathrm{ini}}$	The initial energy of node.
$d_{i,j}$	The transmission distance from the node $n_{i}$ to its neighbor $n_{j}$.
$\theta_{i,j}$	The direction angle between the node $n_{i}$ and $n_{j}$ towards the sink node.
$\varepsilon_{i,j}$	The residual energy of neighbor $n_{j}$$\;\mathrm{of}\;n_{i}$.
e	Euler’s constant. Approx. 2.71828.
D	The network density.
$F_{i}$	The forwarding zone for the node $n_{i}$
$w_{i}$	The width of forwarding zone for the node $n_{i}$
A	The area of the target field.
r	The communication range of node.
$Z_{i}$	The candidate forwarding zone of $n_{i}$.
C(i)	The candidate forwarding set of $n_{i}$.
$\alpha_{\theta },\alpha_{d},\alpha_{\varepsilon }$	The control parameters of routing metrics.
${\bar{Q}}_{i,j}$	The routing quality of neighbor $n_{j}$ of $n_{i}$.

**Table 3:** Simulation Parameters.

Parameters	Values
Network topology	Random
Deployment area	300 m×50 m
Generate rate	1 packet/0.1 s
Number of nodes	200–300
Sink	1 static sink (edge)
Source node	1 static node (random)
Transmission rate	1 Mbps
Communication range	40 m
Simulation time	2000 s
RF channels	2.4 GHz
Packet size Initial energy Sleep power	1024 bits 0.5 J 0.78 mW
$E_{\mathrm{elec}}$ $\varepsilon_{\mathrm{amp}}$ $\varepsilon_{\mathrm{fs}}$	50nJ/bit $0.0013\mathrm{pJ}/\mathrm{bit}/m^{4}$ $10\mathrm{pJ}/\mathrm{bit}/m^{2}$
$\alpha_{\theta },\alpha_{d},\alpha_{\varepsilon }$	1,1,1

## Data Availability

Not applicable.
